# Association of the NHS Health Check with all-cause mortality: a longitudinal cohort study in primary care

**DOI:** 10.3399/BJGP.2025.0451

**Published:** 2026-08-11

**Authors:** Jasjot Saund, Hiten Dodhia, Jacopo Vanoli, Pierre Masselot, Stephen O’Neill

**Affiliations:** 1 London Borough of Lambeth Public Health Team, London, UK; 2 London School of Hygiene and Tropical Medicine, London, UK; 3 Faculty of Life Sciences & Medicine, School of Life Course & Population Health Sciences, Department of Population Health Sciences, King’s College London, London, UK; 4 Institute of Social and Preventive Medicine, University of Bern, Bern, Switzerland

**Keywords:** cardiovascular diseases, London, longitudinal studies, population health, primary health care, risk reduction

## Abstract

**Background:**

Cardiovascular disease (CVD) prevention is a policy priority because of widening inequalities and increasing preventable deaths. The NHS Health Check (NHS-HC) programme aims to promote early detection and prevention of CVD but has a wider-reaching impact owing to targeting multiple risk factors. Although programme evaluations demonstrate improvements in detection and surrogate markers, research on mortality outcomes remain limited, particularly using representative, real-world data.

**Aim:**

To evaluate the effectiveness of NHS-HCs in reducing all-cause mortality.

**Design and setting:**

Longitudinal cohort study in an inner-city London borough.

**Method:**

Data from electronic primary care records were analysed for 158 645 eligible participants (2009–2023). Survival was modelled using a Cox proportional hazards model with time-varying exposure. All-cause mortality risk was compared between those attending ≥1 NHS-HC (*n* = 42 589) and those who did not (*n* = 116 056). Sociodemographic, geographic, and behavioural factors were adjusted using propensity score weighting.

**Results:**

Of the 158 645 participants, 63 347 (39.9%) were of Black, Asian, mixed, or other ethnicity, and 100 825 (63.6%) lived in the two most deprived quintiles in England. Attendance at ≥1 NHS-HC was associated with lower mortality (adjusted hazard ratio 0.68, 95% confidence interval = 0.63 to 0.74), indicating a 32% reduction in risk. Ten-year absolute mortality risk reduction increased with age, with the largest difference among those aged 70–74 years (18.1% versus 12.9%). Sensitivity analyses supported these findings.

**Conclusion:**

This study suggests significant long-term survival benefit associated with the NHS-HC, supporting continued investment in and optimisation of the programme to provide population health benefits. Findings were robust to missing data assumptions and COVID-19, although residual confounding cannot be ruled out.

## How this fits in

The NHS Health Check (NHS-HC) has been shown to improve detection and management of surrogate markers of health, such as blood pressure and cholesterol. The long-term impact, however, is less evidenced, with mortality reductions seen in a UK Biobank cohort and no mortality reduction seen in a Cochrane review of general health checks, including those outside the UK. The current study added real-world evidence from a diverse UK urban population, and a 15-year study period. Survival analysis of GP electronic health record data shows reduced mortality in those who attended an NHS-HC compared with those who did not. These findings should be interpreted cautiously given the observational nature of the study design.

## Introduction

Cardiovascular diseases (CVDs) remain an important public health issue contributing to worsening health inequalities. An increase in CVD-related deaths over several years^1^ and an increasing burden on the NHS because of morbidity has made CVD a priority for prevention and early management in the UK.^2^ The NHS Health Check (NHS-HC) programme, a population-level preventive initiative, aims to identify individuals at high risk of developing CVD. The programme integrates with existing clinical pathways to allow appropriate follow-up for those identified who require intervention. Since 2009, when it was launched, the programme has evolved to maximise efficiency and cost-effectiveness.^3^

Currently, NHS-HCs are a statutory responsibility for local authorities to commission.^4^ Eligible individuals aged 40–74 years without pre-existing CVDs are invited to a check every 5 years where their risk of an acute cardiovascular event is estimated from a combination of lifestyle factors, physical measurements, and biochemical tests.^4,5^ Participants are stratified by risk, offered tailored interventions to support lifestyle change for behavioural risk factors, and prescribed medical management where required. Inherently this approach has an impact on the risk of a range of conditions and thus extends beyond CVD to influence overall mortality.^6^

There was speculation about the programme’s effectiveness at inception owing to implementation without gold-standard randomised controlled trial (RCT) evidence.^7^ Many studies have since evaluated its clinical- and cost-effectiveness.^8,9^ As a result of the long latency between intervention and measurable outcomes, these have relied on surrogate outcomes^10,11^ and have shown a predominantly positive picture of the NHS-HC in reducing cholesterol levels and blood pressure, and the early detection of disease.^12^ However, the effectiveness of NHS-HCs is multifaceted,^13^ shaped by the check itself, uptake,^14^ communication strategies,^15^ administrative delivery,^13^ prioritisation, and follow-up care.^16,17^ Variation in how the administrative aspects of the NHS-HC were carried out by general practices has been shown to have an impact on uptake,^18^ highlighting that it may be predominantly taken up by the ‘worried-well’. This is relevant when evaluating population-level outcomes such as mortality, where differences in uptake may influence observed survival benefits. Although various components of the programme have been evaluated individually, it is difficult to synthesise these into a coherent understanding of the NHS-HCs’ overall population-level impact.

Other evaluations have shown lower than expected NHS-HC attendance in the first 4 years.^18,19^ Those who did attend showed an increase in risk-factor detection,^11,20^ associated with newly prescribed statin and anti-hypertensive medications.^18^ A difference-in-differences analysis between 2009 and 2013 was one of the first to quantify the impact of these increases in detection and prescriptions of preventive measures.^20^ Although the study found statistically significant reductions in outcomes such as cardiovascular risk score, blood pressure reading, and cholesterol levels, the absolute reductions were small and the overall attendance rates were below the national targets set out to reach cost-effectiveness.

Recent findings from a government-commissioned review of the NHS-HC reaffirm its role in identifying modifiable risk factors, with approximately 75% of attendees presenting with ≥1 such factors. This finding contrasts with previous concerns that the programme disproportionately attracts the ‘worried-well’.^3^ A retrospective matched case–control study has reported significant reductions in CVD and all-cause mortality over an average follow-up of 9 years.^6^ Although this study demonstrates impressive outcomes, it is limited by its focus on a non-representative cohort that was 96% White, who are likely subject to healthy participant bias.

These limitations emphasise the importance of rigorously evaluating NHS-HCs as they are delivered in practice. This is particularly when they are embedded within clinical pathways, target apparently healthy individuals, and are intended to improve disease detection,^6,11,18,20,21^ subsequent interventions,^18,22,23^ and risk-factor control.^20^ The current study presents an analysis of the association of the NHS-HC programme with all-cause mortality in an inner-city local authority in the UK.

## Method

### Data source

This study utilised primary care electronic health records (EHRs) covering all patients who registered with Lambeth GPs between 2005 and 2023 (>1 million individuals). This included individuals who were actively registered at the time of data extraction as well as those who had previously deregistered for any reason. Data extraction was performed using SNOMED-CT codes.

### Study design and population

This retrospective cohort study adheres to Strengthening the Reporting of Observational Studies in Epidemiology guidelines.^24^ The study window began on 1 January 2009 (when the NHS-HC programme was implemented) and ended on 31 August 2023 (the date of data extraction). The study population was selected based on eligibility for an NHS-HC within the study window.^25^ This included those aged between 40 and 74 years, and excluded anyone with coded history of: ischaemic heart disease, myocardial infarction, chronic kidney disease, hypertension, atrial fibrillation, transient ischaemic attack, heart failure, peripheral arterial disease, stroke, ever being prescribed statins, or ever having a QRISK score >20%. To ensure data currency, eligible participants were required to be registered with Lambeth primary care with a minimum 1-year lookback period in EHRs.

### Follow-up and censoring

Follow-up commenced at index date (time zero), defined as the date participants became eligible for NHS-HCs: either on their 40th birthday or registration date. Birth dates were assumed to be 1 January of the recorded year of birth for each participant. Follow-up concluded at the earliest occurrence of one of: death (event), loss to follow-up (such as, migration), or administrative censoring at the study endpoint (31 August 2023). Time to event was calculated as the interval between the index date and the end date, with censoring assumed to be non-informative in accordance with survival analysis methodologies.^26^

### Exposure and outcome definitions

Exposure was defined as NHS-HC attendance where the date of the coded entry was used as the date of exposure. NHS-HC attendance SNOMED-CT codes reflect those used in national data capturing. The outcome was defined as all-cause mortality as registered in EHRs, with date of death as the event date.

### Covariates

Baseline demographic, clinical, and behavioural characteristics were defined. These included a natural spline of age with two degrees of freedom, sex (male or female), ethnicity (census 2001 high-level categories), Index of Multiple Deprivation (IMD) quintile, smoking status (current smoker, former smoker, or never smoker), units of alcohol consumed per week (none, 1–14, 15−35, or >35), primary care network (derived from general practice code), lower-layer super output area, main spoken language (English or non-English), enrolment period (2009–2011, 2012–2014, 2015–2017, 2018–2020, or 2021–2024), family history of coronary heart disease (CHD) (yes or no), history of cancer (yes or no), history of serious mental illness (yes or no), history of rheumatoid arthritis (yes or no), history of systemic lupus erythematous (yes or no), history of liver disease (yes or no), and body mass index (BMI) category (as per World Health Organization weight categories). For BMI and alcohol consumption, the baseline value was defined as the closest recorded measurement to the index date within a ±10-year window. For smoking status, current smoker (at baseline) was defined as the presence of a current smoking code within a ±10-year window. Never smoker was defined as an absence of any smoking codes, and former smoker defined as presence of a current smoking code but not within 10 years of the index date. For all other variables, baseline was defined as the recorded status at the time of the index date. Missing data patterns were evaluated, and approaches are discussed in Supplementary Box S1, with per cent missingness shown in Supplementary Table S1.

### Statistical analysis

Baseline characteristics were summarised using descriptive statistics, stratified by NHS-HC status and age. Differences between groups were assessed using appropriate χ^2^-tests in all cases as only categorical variables were used.

The primary analysis employed a time-varying Cox proportional hazards model to examine the association between NHS-HC attendance and all-cause mortality. At index date (time zero), all participants were classified as unexposed (NHS-HC = 0). Participants who subsequently attended an NHS-HC were reclassified as exposed (NHS-HC = 1) from the date of attendance onwards. Modelling NHS-HC attendance as a time-varying covariate avoided immortal time bias, as person–time before attendance contributes to the unexposed category rather than being incorrectly attributed to the exposed group.^27^ This approach also mitigates the need for artificial time alignment of exposed and unexposed participants, which would otherwise require additional assumptions and participant matching, thereby reducing the available analytical sample. Total weighted person–years were calculated for each exposure group. Consistent with the time-varying Cox model, individuals were allowed to contribute person–time to >1 exposure group over the follow-up period.

A secondary analysis assessed the association between NHS-HC attendance and premature (aged <75 years) all-cause mortality. This approach used the same time-varying Cox proportional hazards model where follow-up time was additionally right censored if participants reached 75 years old.

All models were adjusted for all baseline covariates via propensity score weighting (see Supplementary Box S2). Weighted population characteristics are show in Supplementary Tables S2 and S3 (stratified by age). As well as adjusting for measured confounders, *E*-values were calculated to estimate the potential impact of unmeasured confounding. Formula 1 was applied as per the methods from VanderWeele *et al*:^28^


$$\text{Estimate: }E\text{-value}=RR^{*}+\sqrt{\left\{RR^{*}\times (RR^{*}-1)\right\}}$$

$$\text{Upper limit: }E\text{-value}=UL^{*}+\sqrt{\left\{UL^{*}\times (UL^{*}-1)\right\}}$$

RR is the risk ratio; RR* is the inverse of the RR; UL is the upper limit of the confidence interval (CI); and UL* is the inverse of the UL.

Absolute risk of death per age category was calculated using the same weighted Cox proportional hazards model with an age stratification term, allowing the baseline hazard to differ by age. The model was used to calculate the baseline survival probability per age group at 10 years from the index date, where absolute risk for the no NHS-HC group is equal to 1 minus the baseline 10-year risk, and absolute risk for the NHS-HC group is 1 minus the baseline 10-year risk to the power of the hazard ratio (HR). The risk difference is the difference between the two.

Age-stratified adjusted HRs were calculated by including an interaction term between exposure and age, and are reported in Table 1.

**Table 1.** Absolute risk of death after 10 years from index date stratified by exposure group and 5-year age category^a^

			Predicted 10-year absolute risk of death		
**Index age, years**	**Patients,** ***n*** **^b^**	**Deaths,** ***n*** **^b^**	**No NHS-HC group, %**	**NHS-HC group, %**	**Adjusted HR (95% CI)**
**40–44**	99 300	784	1.2	0.8	0.79 (0.66 to 0.96)
**45–49**	24 268	591	2.2	1.5	0.67 (0.54 to 0.84)
**50–54**	15 167	591	3.6	2.5	0.71 (0.57 to 0.88)
**55–59**	9150	509	5.0	3.5	0.76 (0.62 to 0.95)
**60–64**	5850	527	7.4	5.4	0.72 (0.58 to 0.88)
**65–69**	3117	461	12.5	8.8	0.57 (0.43 to 0.75)
**70–74**	1793	414	18.1	12.9	0.54 (0.39 to 0.74)

^a^Adjusted HRs in age interaction model. ^b^ *n = total number of individuals in each age category (unweighted count); Deaths = total observed deaths in that age category over the full study period (unweighted, not split by exposure group, since NHS-HC attendance is time-varying). No NHS-HC and NHS-HC columns show the model-predicted 10-year absolute risk of death from the weighted, propensity-adjusted, age-stratified Cox model, using the pooled adjusted HR; they are not equal to Deaths ÷ n, which reflects crude, unadjusted mortality over variable follow-up time rather than a fixed 10-year horizon. HR = hazard ratio. NHS-HC = NHS Health Check.*

The proportional hazards assumption was tested using Schoenfeld residuals. If violations were detected, the model was stratified into three 5-year strata, with HRs calculated separately for each stratum.

To explore effects of imputation on the analysis, the primary analyses were carried out on both a complete-case dataset and multiple imputation by chained equations (MICE) datasets. All weighted datasets were validated against the GP-registered population of those eligible for NHS-HCs (see Supplementary Box S3).

All statistical analyses were performed using R (version 4.4.2).

### Subgroup analysis

The primary analysis was repeated on 10 subgroups consisting of the five IMD quintiles and the five high-level ethnicity categories.

## Results

### Descriptive results

Of the 158 645 participants analysed, 42 589 received ≥1 NHS-HC and 116 056 received none (Table 2). There was a total of 3877 deaths (2.4%), 761 (1.8%) in the NHS-HC group and 3116 (2.7%) in the no NHS-HC group. Premature deaths accounted for 3099 deaths, with 620 (1.5%) in the NHS-HC group and 2479 (2.1%) in the no NHS-HC group. The total weighted person–years of follow-up was 2 343 373, with 1 660 913 weighted person–years in the no NHS-HC group and 681 460 weighted person–years in the NHS-HC group.

**Table 2.** Baseline characteristics compared between NHS-HC and no NHS-HC group in dataset before weighting

Characteristic^a^	Overall,*n* = 158 645	No NHS-HC group,*n* = 116 056	NHS-HC group,*n* = 42 589	*P*-value
**Year of birth, mean (SD)**	1968.69 (9.92)	1969.34 (10.04)	1966.92 (9.36)	<0.001
**Sex, female**	76 526 (48.2)	55 315 (47.7)	21 211 (49.8)	<0.001
**IMD quintile**				<0.001
1 (most deprived)	29 065 (18.3)	21 582 (18.6)	7483 (17.6)	
2	71 760 (45.2)	52 738 (45.4)	19 022 (44.7)	
3	44 136 (27.8)	31 875 (27.5)	12 261 (28.8)	
4	11 414 (7.2)	8211 (7.1)	3203 (7.5)	
5 (least deprived)	2270 (1.4)	1650 (1.4)	620 (1.5)	
**Smoking status**				0.038
Never	106 151 (66.9)	77 444 (66.7)	28 707 (67.4)	
Former	3428 (2.2)	2530 (2.2)	898 (2.1)	
Current	49 066 (30.9)	36 082 (31.1)	12 984 (30.5)	
**Enrolment**				<0.001
2009–2011	67 159 (42.3)	44 086 (38.0)	23 073 (54.2)	
2012–2014	23 992 (15.1)	17 018 (14.7)	6974 (16.4)	
2015–2017	23 009 (14.5)	17 121 (14.8)	5888 (13.8)	
2018–2020	23 599 (14.9)	19 542 (16.8)	4057 (9.5)	
2021–2024	20 886 (13.2)	18 289 (15.8)	2597 (6.1)	
**Family history of CHD**	2401 (1.5)	978 (0.8)	1423 (3.3)	<0.001
**Ethnicity**				<0.001
White	95 298 (60.1)	70 575 (60.8)	24 723 (58.1)	
Black	37 345 (23.5)	25 997 (22.4)	11 348 (26.6)	
Asian	11 491 (7.2)	8571 (7.4)	2920 (6.9)	
Mixed	7136 (4.5)	5368 (4.6)	1768 (4.2)	
Other	7375 (4.6)	5545 (4.8)	1830 (4.3)	
**Primary care network**				<0.001
1	13 699 (8.6)	7422 (6.4)	6277 (14.7)	
2	11 164 (7.0)	8211 (7.1)	2953 (6.9)	
3	25 378 (16.0)	19 787 (17.0)	5591 (13.1)	
4	25 069 (15.8)	18 356 (15.8)	6713 (15.8)	
5	12 269 (7.7)	9118 (7.9)	3151 (7.4)	
6	22 320 (14.1)	17 303 (14.9)	5017 (11.8)	
7	13 297 (8.4)	9960 (8.6)	3337 (7.8)	
8	15 751 (9.9)	11 983 (10.3)	3768 (8.8)	
9	19 698 (12.4)	13 916 (12.0)	5782 (13.6)	
**BMI**				<0.001
Normal weight	69 505 (43.8)	51 451 (44.3)	18 054 (42.4)	
Obese	30 932 (19.5)	22 628 (19.5)	8304 (19.5)	
Overweight	55 225 (34.8)	39 663 (34.2)	15 562 (36.5)	
Underweight	2983 (1.9)	2314 (2.0)	669 (1.6)	
**Alcohol, units per week**				<0.001
None	48 601 (30.6)	34 972 (30.1)	13 629 (32.0)	
>35	3393 (2.1)	2464 (2.1)	929 (2.2)	
1–14	93 051 (58.7)	69 066 (59.5)	23 985 (56.3)	
15–35	13 600 (8.6)	9554 (8.2)	4046 (9.5)	
**Index age, years**				<0.001
40–54	138 735 (87.4)	101 730 (87.7)	37 005 (86.9)	
55–64	15 000 (9.5)	10 375 (8.9)	4625 (10.9)	
65–74	4910 (3.1)	3951 (3.4)	959 (2.3)	
**Cancer**	2742 (1.7)	2026 (1.7)	716 (1.7)	0.394
**Serious mental illness**	3245 (2.0)	2441 (2.1)	804 (1.9)	0.008
**Rheumatoid arthritis**	617 (0.4)	432 (0.4)	185 (0.4)	0.086
**Systemic erythematous lupus**	213 (0.1)	155 (0.1)	58 (0.1)	0.961
**Liver disease**	728 (0.5)	559 (0.5)	169 (0.4)	0.030
**Main spoken language not English**	37 322 (23.5)	28 068 (24.2)	9254 (21.7)	<0.001

^a^Data are *n* (%) unless otherwise specified. BMI = body mass index. CHD = coronary heart disease. IMD = Index of Multiple Deprivation. NHS-HC = NHS Health Check. SD = standard deviation.

Table 2 presents the counts and percentage distributions of categorical variables across each exposure group before weighting. Overall, 39.9% of the cohort were of Black, Asian, mixed, or other ethnicity (41.9% among the NHS-HC group and 39.2% among the no NHS-HC group), and 63.6% of the cohort lived in the two most deprived IMD quintiles. NHS-HC attendees were noted to include more female patients (49.8% versus 47.7%) and fewer people living in the most deprived quintile (17.6% versus 18.6%). Differences in smoking and drinking alcohol were small but showed that NHS-HC attendees included fewer people who smoked (30.5% versus 31.1%), more people who drank >14 units of alcohol per week (11.7% versus 10.3%), but fewer people overall who drank alcohol (68.0% versus 69.9%). NHS-HC attendees also included more people who were overweight (36.5% versus 34.2%), as well as more people with a family history of CHD (3.3% versus 0.8%). Baseline characteristics were taken at a 10-year window from the index date and thus NHS-HC attendees may have had baseline characteristics taken after their NHS-HC.

### Propensity score weighting results

Standardised mean differences (SMDs) between NHS-HC and no NHS-HC groups were calculated before and after propensity score weighting. Figure 1 shows the SMDs before and after weighting. All variables after weighting had an acceptable SMD of <0.1.

**Figure 1.**
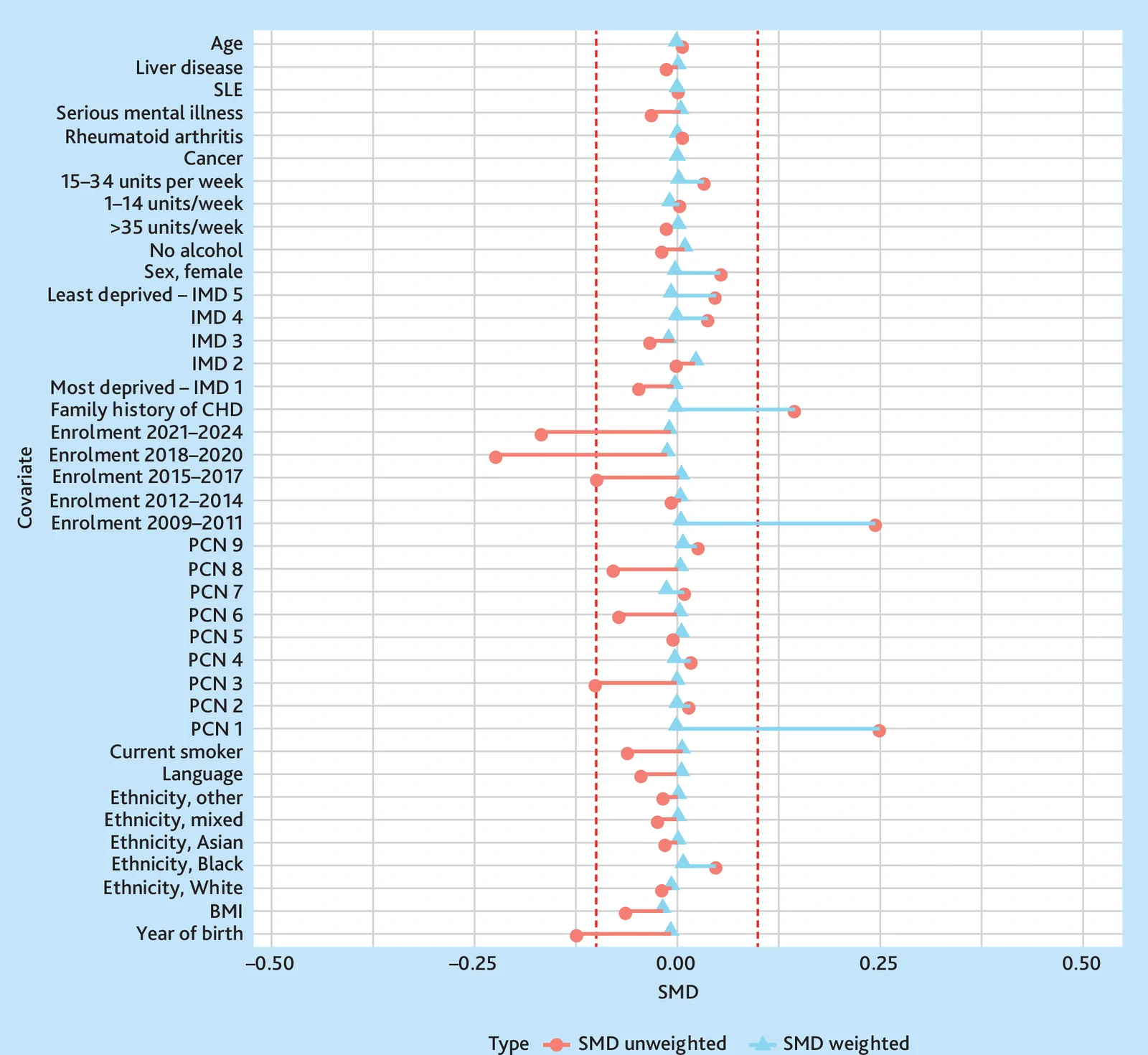
Standardised mean differences (SMDs) between exposure groups for each variable before (red) and after (blue) applying weighting. Red dashed line indicates 0.1 SMD. BMI = body mass index. IMD = Index of Multiple Deprivation. PCN = primary care network. SLE = systemic erythematous lupus.

### Survival analysis results

For the primary analysis investigating the association with all-cause mortality, adjusted HRs for NHS-HCs of 0.68 (95% CI = 0.63 to 0.74) highlight that NHS-HCs were statistically significantly associated with a 32% reduced risk of death (data not shown). Schoenfeld residuals indicated proportional hazard’s assumption held for the exposure (Schoenfeld test *P* = 0.55). Figure 2 presents the estimated survival curves from the Cox model, comparing those who attended an NHS-HC with those who did not. Calculated *E*-values were 2.30 for the estimate and 2.04 for the upper limit of the CI (closest to null).

**Figure 2.**
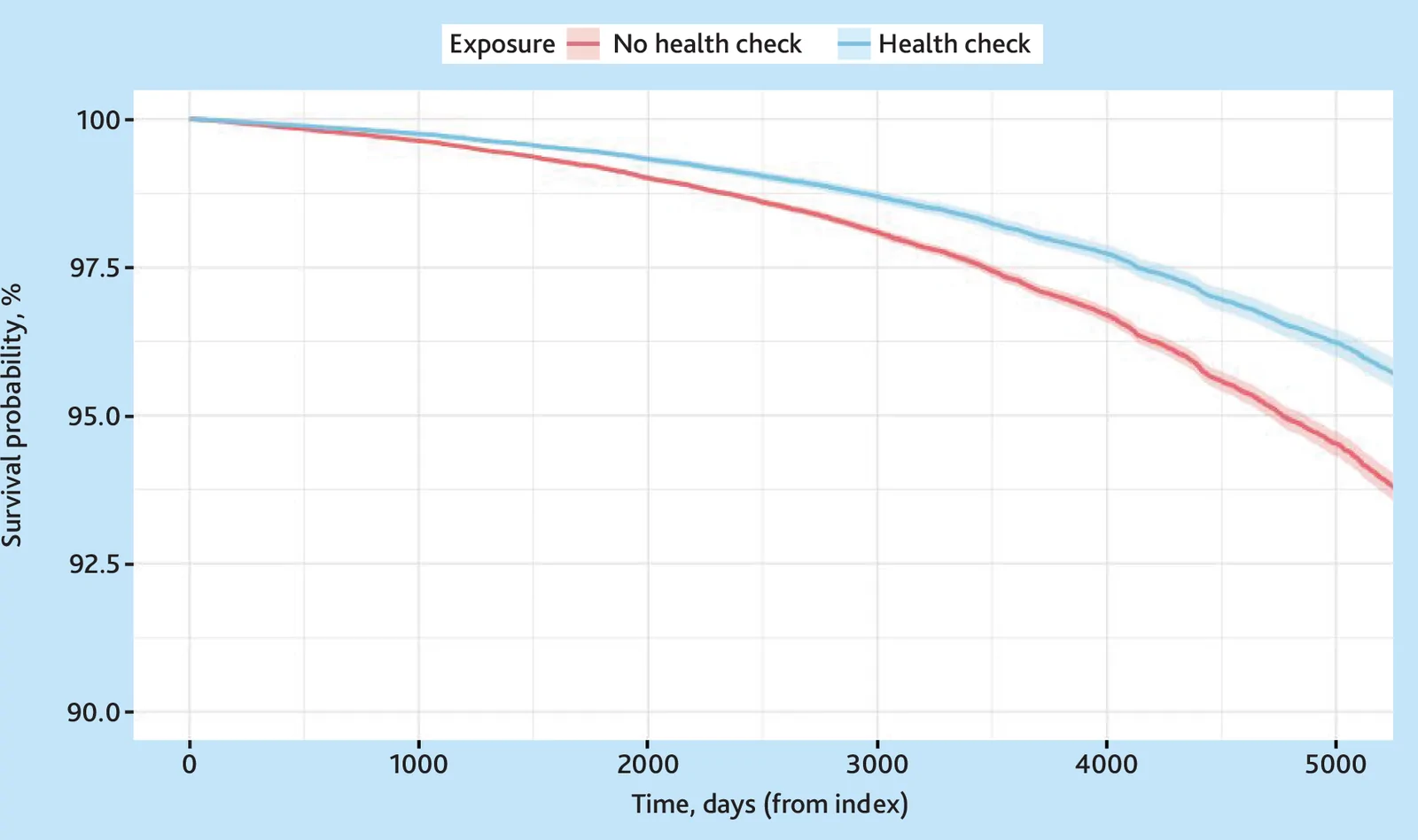
Estimated adjusted survival curve from weighted Cox proportional hazards model. Baseline covariates balanced include: sex; body mass index; ethnicity; main spoken language; smoking status; primary care network; enrolment period; family history of chronic heart disease; Index of Multiple Deprivation quintile; age; alcohol intake; and history of cancer, rheumatoid arthritis, serious mental illness, systemic lupus erythematous, or liver disease.

An estimated absolute risk of death at 10 years was calculated from the survival model, stratified by age, with the most notable reduction observed in individuals aged ≥65 years who had ≥1 NHS-HC, where risk decreased from 12.5% to 8.8% for those aged 65–69 years and from 18.1% to 12.9% for those aged 70–74 years (Table 1).

The secondary model investigating the association of NHS-HCs with premature all-cause mortality revealed an adjusted HR for NHS-HCs of 0.71 (95% CI = 0.65 to 0.78); Schoenfeld residuals indicated that proportional hazard’s assumption held for the exposure (Schoenfeld test *P* = 0.65) (data not shown).

Subgroup analyses are reported in Supplementary Table S4 and highlight a similar positive impact of NHS-HCs on all-cause mortality across all subgroups. If a significant effect is only seen in IMD quintiles 1, 2, and 3, and White, mixed, and other ethnicity groups, this is mostly related to large populations in these subgroups, allowing for less uncertain estimates.

Sensitivity analysis supported the robustness of the main findings (see Supplementary Box S4).

## Discussion

### Summary

This study assessed the association of NHS-HCs with all-cause mortality in an ethnically diverse population, representative of an inner-city London borough. Although previous studies have shown NHS-HCs can benefit the population through early detection of CVDs and improved surrogate biomarkers, evidence about the impact on survival has been minimal and contested.^7^

This analysis, adjusting for a range of confounding factors, suggests a significant reduction in the risk of all-cause mortality and premature mortality among participants attending ≥1 NHS-HC. With a large sample size and extended follow-up, the current study has provided evidence of the programme’s long-term effectiveness in improving survival outcomes in the real world.

### Strengths and limitations

Key strengths of the dataset include its large sample size, ethnic diversity, representation of socioeconomically deprived and vulnerable populations, an extended follow-up period, and real-world representativeness. Subgroup population effects were limited by smaller sample sizes and thus reduced strength of these findings.

As an observational study, potential residual confounding remains a limitation. Despite adjustments for a wide range of covariates, unmeasured factors, particularly the risk of self-selection bias, may still in part influence outcomes. Self-selection bias is a type of selection bias describing the effect of voluntary participation in the NHS-HC programme, based on characteristics such as being more health aware. This in theory can be adjusted for and is distinct from the healthy volunteer bias that is observed when the whole sample is subject to a selection bias, such as that seen in the UK Biobank population.^6^ In the current study the population is representative of the target population as evidenced by the validation.

Previous research exploring self-selection bias in general health checks through use of negative control outcomes found a significant reduction in all-cause mortality after adjustment similar to that in the present study (27%). However, the authors of this research argue that, because a significant association was found in both lifestyle-related mortality and the negative control outcome (non-lifestyle-related mortality), this indicates the presence of residual confounding.^29^

Although the use of negative control outcomes can help detect residual confounding, this approach has important limitations. The classification of deaths as ‘lifestyle-related’ versus ‘non-lifestyle-related’ is inherently ambiguous, as many causes of death are influenced by a complex interplay of behavioural, biological, and social factors. Consequently, identifying a truly independent negative control outcome is challenging. Furthermore, even with extensive covariate adjustment, adjustments for physical health were limited to hospital discharge codes only,^29^ which do not reflect cardiovascular conditions managed solely in primary care or outpatient settings. This limited granularity can lead to underestimation of baseline comorbidity and incomplete adjustment for differences in health and behaviours between participants and non-participants, thereby leaving potential bias unaccounted for. The current study did not include participants with pre-existing CVD as per the eligibility criteria for NHS-HCs; however, the authors of the current study recognise the limitation of unmeasured confounding factors, including those contributing to a potential self-selection bias. The authors further recognise the limitation of the current dataset that does not reliably capture cause of death.

To address this concern, the current study attempted to quantify the unmeasured confounding to estimate its likelihood for fully explaining the observed association. *E*-value calculations suggest that an unmeasured confounder would need to be associated with both NHS-HC attendance and mortality with a risk ratio of at least 2.04, beyond that of measured confounders, to fully account for the observed association. Taken together, although some residual confounding cannot be excluded, it is unlikely that residual unmeasured confounding, requiring a confounding strength double that of already measured factors, fully explains the observed mortality decrease benefit seen in this study.

Additionally, although the use of all-cause mortality as the primary outcome may limit the ability to detect specific effects on cardiovascular mortality, a primary target of the NHS-HC programme, it also represents a key strength. Unlike surrogate markers, all-cause mortality provides a definitive, clinically meaningful endpoint that captures the net impact of the intervention, including both intended and unintended effects.

A key analytical strength of this study is the use of advanced covariate balancing techniques (inverse probability of treatment weighting) to reduce bias and approach causal inference. This method, compared with matching, made it possible to consider a larger dataset, enhancing statistical power. The exposure was modelled as time varying to reflect changes in participants' exposure status over the follow-up period. Although covariates may change during the follow-up period, in this study the primary interest was in adjusting for confounding that occurs before exposure assignment, rather than post-exposure covariate shifts. The assumption of time-fixed confounding therefore remains appropriate for the purposes of this analysis.

Although missing data presented a potential challenge, MICE was employed alongside mode imputation and complete-case analysis to assess its impact. Notably, results from the imputed datasets showed minimal variability, suggesting the findings were robust to different assumptions about missing data. Complete-case analyses further supported the consistency of the results.

### Comparison with existing literature

The current findings contribute to the ongoing debate regarding the effectiveness of NHS-HCs, particularly in reducing mortality. A recent observational study by McCracken *et al* reported substantial reductions in all-cause mortality following NHS-HC participation.^6^ However, this study was limited by the well-documented biases inherent to the UK Biobank dataset where participants are typically more health conscious, introducing healthy attender bias. This may make them more receptive to NHS-HC interventions and more likely to benefit from earlier diagnoses than the general population. Although this highlights the benefit of the NHS-HC in a receptive population, it does not reflect the potential real-world conditions where behavioural innervations may have less of an impact in populations that are less amenable. Additionally, their study comprised a predominantly White cohort (96%) and from less deprived backgrounds, with 29% falling above the UK median for Townsend deprivation scores.^6^ As a result, the findings may not be generalisable to more deprived and ethnically diverse populations.

Other notable studies include the Inter99 study, a large-scale RCT assessing non-pharmacological lifestyle interventions for the prevention of ischaemic heart disease and stroke.^30,31^ This study, along with 14 other RCTs, was included in a Cochrane review comparing general health checks with no health checks in adults unselected for disease or risk factors and found no clear benefit in reducing morbidity or mortality.^31,32^ Both analyses share key limitations when compared with the current study investigating the effect of the NHS-HC programme. In both the Inter99 study and the Cochrane review, participants included individuals with existing comorbidities, and interventions focused on behavioural counselling without pharmacological risk-factor management. It is therefore plausible that, in cohorts without established CVD at baseline, a combined strategy integrating lifestyle modification with targeted pharmacological interventions (such as statins or antihypertensive therapy) would produce greater reductions in cardiovascular risk and event rates. Furthermore, the current findings also highlight the biggest risk reduction in those aged ≥65 years, an age group largely excluded from the RCTs in the Cochrane review. Re-analysis of the data in the Inter99 study using different statistical methods showed a significant difference in all-cause and specific mortality, further highlighting how different statistical methods may answer different research questions.^29,33^

Moreover, the descriptive analysis in the current study showed that, although attendees included slightly more individuals from less deprived backgrounds and fewer people who smoke and who drink alcohol, they were also more overweight and substantially more likely to have a family history of CHD. This pattern suggests that attendees are not uniformly ‘healthier’ than non-attendees.

While acknowledging the findings of the Cochrane review highlighting no impact of non-pharmaceutical general health checks in an undifferentiated population, the current study is specially targeted to seemingly healthy individuals, with pharmacological and non-pharmacological interventions, in a system that is embedded in primary and community care for follow-up.

Although the current study did not assess follow-up actions after an NHS-HC (such as prescriptions or referrals), previous literature presents a mixed picture regarding subsequent management. For example, Robson *et al* reported <10% of those eligible for statins were prescribed them within 12 months,^23^ whereas Debiec *et al* found that, although prescription rates were initially low at 12 weeks post-NHS-HC, they increased significantly over time, particularly in the most high-risk groups, up to at least 18 months.^17^ This pattern may suggest a preference for implementing lifestyle changes in the first instance (as per National Institute for Health and Care Excellence guidance^34^), before initiating pharmacological treatment.

Despite these methodological differences, the authors of the current study accept the possibility of residual confounding from self-selection bias, although argue its impact will be smaller in a subpopulation without pre-existing CVD compared with the general population and is not enough to explain the entire reduced risk found in the present study.

The current findings are in line with McCracken *et al* and additionally add the context of a real-world ethnically diverse cohort.^6^ Where much of the previous literature has focused on surrogate outcomes or process measures (such as rates of diagnosis, prescription, or referral), these endpoints do not always translate into long-term clinical benefit. Use of all-cause mortality in the current study provides a robust and definitive outcome, capturing both intended and unintended effects of the intervention over time. This is especially relevant given the rising premature CVD deaths in the UK. Although the effects of the NHS-HC are not directly comparable with RCTs investigating non-pharmacological effects of general undifferentiated health checks, residual confounding remains a concern and may contribute to, but is unlikely to explain away, the results found in this study.

### Implications for research and practice

The results, when considered alongside previous findings, strengthen the case for continued implementation and optimisation of NHS-HCs, particularly within integrated care systems that support follow-up and long-term management. From a policy perspective, NHS-HCs have the potential to play a pivotal role in addressing health inequalities and reducing premature mortality.

The current testing of digital and workplace-based health checks presents an opportunity to enhance engagement and accessibility. Digital platforms can eliminate traditional barriers to healthcare access by offering flexibility, and workplace initiatives can target underrepresented groups. To fully harness these innovations, their integration into existing frameworks must be strategically planned and rigorously evaluated for effectiveness.

Future research incorporating linked death registry data would allow cause-specific mortality analyses, particularly for cardiovascular mortality, providing clearer insight into the mechanisms underlying any observed survival benefit. Additionally, application of a target trial emulation framework could further strengthen causal inference. This could include an intention-to-treat analysis comparing individuals invited to attend an NHS-HC with those not invited; however, implementation of a true intention-to-treat approach may be challenging in real-world settings where invitation status and timing are not consistently or completely recorded. Alternatively, a per-protocol approach estimating the effect of attendance, while appropriately adjusting for time-varying confounding, may be more feasible within routinely collected data.

Future studies should also focus on extending follow-up periods to better understand the long-term impact of NHS-HCs, particularly within specific subgroups. Intersectional subgroup analyses would provide further insight into potential differential effects across sociodemographic and clinical populations, helping to clarify how benefits may vary in real-world settings.

## Data

Raw data are not publicly available owing to the authors' data-sharing agreements with Lambeth GPs.
